# Cell Sizes Matter for Industrial Bioproduction, a Case of Polyhydroxybutyrate

**DOI:** 10.1002/advs.202412256

**Published:** 2025-02-18

**Authors:** Yi‐Ling Chen, Xu Liu, Li‐Zhan Zhang, Ji‐Shuai Yang, Wei‐Ke Guo, Shuang Zheng, Jia‐Le Wang, Fu‐Qing Wu, Xu Yan, Qiong Wu, Guo‐Qiang Chen

**Affiliations:** ^1^ School of Life Sciences Tsinghua University Beijing 100084 China; ^2^ Tsinghua‐Peking Center for Life Sciences Tsinghua University Beijing 100084 China; ^3^ PhaBuilder Biotechnology Co. Ltd. Shunyi District Beijing 101309 China; ^4^ State Key Laboratory of Animal Nutrition Institute of Animal Science Chinese Academy of Agricultural Sciences Beijing 100193 China; ^5^ Key Lab of Industrial Biocatalysts of the Ministry of Education Department of Chemical Engineering Tsinghua University Beijing 100084 China; ^6^ Center for Synthetic and Systems Biology Tsinghua University Beijing 100084 China

**Keywords:** cell sizes, ClpXP, Halomonas, morphology engineering, mreB, next generation industrial biotechnology, PHB

## Abstract

Most bacterial cells are 1–2 microns in size, limiting intracellular products like polyhydroxyalkanoates (PHA) accumulation. Cell size is regulated by key genes such as *mreB* and *minCD*, which encode cellular skeleton protein and control cell fission ring location, respectively. Their expression changes significantly affect microbial growth. This study successfully redesigns the ClpXP protein degradation system by deleting the *sspB* gene and using mutated SsrA tags with different degradation rates to control MreB degradation. Dynamic degradation of MreB allows non‐model bacterium *Halomonas bluephagenesis* to grow normally and increase cell size simultaneously. Combined with overexpression of *minCD*, *H. bluephagenesis* with progressive MreB degradation increases the cell size further, albeit with a reduced growth rate. *H. bluephagenesis* CYL0307, with the PHB granule‐associated protein PhaP1 deleted and *phaAB_Re_* overexpressed in the MreB‐degraded strain, increases cell volume more than nine times compared to the original strain. CYL0307 produces 149 g L^−1^ cell dry weight containing 82% PHB after 44 h in a 5000 L bioreactor, with cells containing single large PHB granules, simplifying recovery and purification. These results provide a post‐translational gene regulation method in *H. bluephagenesis* and a strategy for enhancing PHB production via morphological engineering.

## Graphical abstract

Most bacterial cells are small, restricting the accumulation of intracellular products like polyhydroxyalkanoates (PHA). To solve this problem, the ClpXP system in *Halomonas bluephagenesis* is identified and redesigned. Then MreB is degraded progressively by this engineered system, expanding cell size more than nine times. Enlarged cells enhance the production of PHA and bring many advantages for downstream extraction.

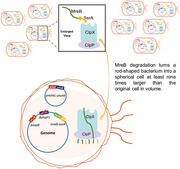

## 1 Introduction

Bacteria are widely used as cell factories in bioproduction processes. However, most bacterial cells have small sizes of ≈1 µm, limiting the available space for accumulating intracellular products, such as polyhydroxyalkanoates (PHA).^[^ ^1^, ^2^, ^3^ ^]^ Therefore, engineering cell size (morphology engineering) is crucial to increase cellular space and enhance the accumulation of intracellular bioproducts.^[^ ^2^, ^4^, ^5^, ^6^ ^]^ Cell sizes are regulated by several key genes including *mreB* and *minCD*, which encode proteins related to the maintenance of rod shape and division of bacteria, respectively.^[^ ^7^, ^8^, ^9^ ^]^ Deletion of *mreB* gene in *E. coli* transforms rod‐shaped bacteria to spherical ones while altering the expression level of *minCD* elongates cells.^[^ ^4^ ^]^ However, all these manipulations may negatively affect cell growth, resulting in decreased cell dry weights (CDW) and reduced production.^[^ ^2^, ^4^, ^5^ ^]^ Methods to regulate the levels of cytoskeletal proteins like MreB in living cells are essential for engineering cell sizes while maintaining normal growth.

Many molecular biology tools have been developed for down‐regulating protein levels, such as Clustered Regularly Interspaced Short Palindromic Repeat interference (CRISPRi) and small RNA interference (RNAi). ^[^ ^10^, ^11^, ^12^ ^]^ These methods regulate the transcription and translation processes, thus modulating protein expression levels in their generation phase. Besides, these methods cannot influence proteins that have already been expressed, nor can they regulate gene expression during the stationary phase, when both transcription and translation are significantly reduced.^[^ ^13^ ^]^ On the other hand, when essential genes related to cell growth are down‐regulated, the whole system is required to be carefully induced to avoid poor growth. Both the optimization of induction time and concentrations of the inducer increase the complexity of these tools and limit their industrial applications.

**Figure 1:**
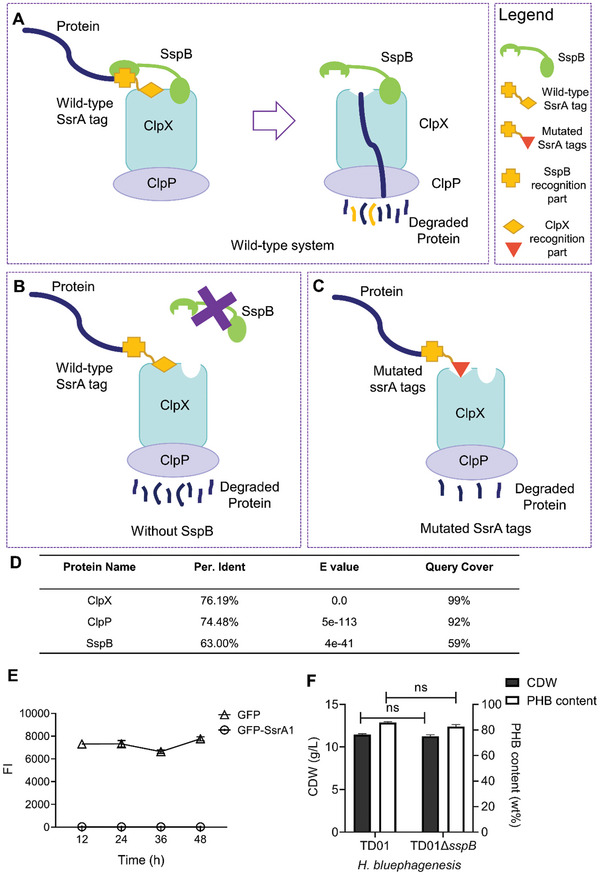
Identification of the ClpXP system and its engineering in *H. bluephagenesis*. A) the wild‐type ClpXP system in *H. bluephagenesis*. B,C) Schematic diagram of the construction of the engineered ClpXP system. D) The protein BLAST results of ClpX, ClpP, and SspB between *Halomonas bluephagenesis* TD01 and *E. coli*. Per. Ident, E value, and query cover were obtained via the NCBI website (https://blast.ncbi.nlm.nih.gov/Blast.cgi). E) Fluorescence intensity (FI) was measured by flow cytometry. GFP and GFP fused with SsrA tags were expressed in plasmids in *H. bluephagenesis* TD01, respectively. F) Cell dry weight (CDW) and PHB contents produced by *H. bluephagenesis* TD01 and CYL0119 (TD01Δ*sspB*), grown in 60 MM for 48 h, respectively. All data represent the mean of n = 3 biologically independent samples and error bars show s.d. ns indicates *p* >0.05.

In order to overcome these drawbacks, a simple and stable protein down‐regulation approach needs to be developed.^[^ ^13^, ^14^, ^15^ ^]^ In prokaryotes, most proteins are degraded by ATP‐dependent proteases including Clp, Lon, HslUV, and FtsH families.^[^ ^16^, ^17^ ^]^ Among these intracellular proteases, ClpXP is the most well‐studied one for degrading stalled proteins during translation.^[^ ^18^, ^19^ ^]^ When the synthesis of protein is stalled in ribosomes, a transfer‐messenger RNA (tmRNA) is recruited to guide the synthesis of the SsrA tag in the C‐terminal of the stalled protein.^[^ ^20^, ^21^ ^]^ This SsrA‐tagged protein can be recognized and bound to ClpXP complexes, which will unfold and degrade this SsrA‐tagged protein quickly.^[^ ^21^ ^]^ The SsrA tag sequence can be divided into two parts: one part is recognized by ClpXP, and the other one by the SspB protein. During this process, adaptor SspB can recognize and tether SsrA‐tagged protein to ClpXP, assisting in the process of protein degradation (**Figure 1A**).^[^ ^22^ ^]^ Previous studies have shown that in an *E. coli* strain deficient in *sspB*, proteins tagged with the SsrA tag will still be degraded quickly.^[^ ^23^ ^]^ In this study, to enable the engineered ClpXP system to degrade protein at varying rates, the *sspB* gene was deleted to abolish its function for tethering SsrA‐tagged protein to ClpX (Figure **1**B). Next, the ClpX recognition part in the SsrA tag was mutated to weaken the interaction between the ClpX and SsrA tag, resulting in mutated SsrA tags with different degradation rates (Figure 1C).

During this research, *Halomonas bluephagenesis* was chosen for its potential in the next generation industrial biotechnology (NGIB) requiring no sterilization with energy and water saving.^[^ ^24^, ^25^ ^]^ It can naturally accumulate polyhydroxyalkanoates (PHA), which is a family of biopolyesters with many promising applications.^[^ ^26^, ^27^, ^28^, ^29^, ^30^, ^31^ ^]^

This study aims to enlarge the cell size of *H. bluephagenesis* without affecting growth by degrading MreB via redesigning the endogenous protein degradation system ClpXP. In particular, the effects of enlarged *H. bluephagenesis* on cell lysis, cell separation, and PHA production in shake flasks, 7, 100, and 5000 L bioreactor were evaluated. It is the first time that enlarged cells were investigated on how the cell size affected cell growth and poly‐3‐hydroxybutyrate (PHB) production on the industrial scale.

## 2 Results

### 2.1 Identification of ClpXP System and Construction of *H. Bluephagenesis* TD01Δ*sspB*

The intrinsic ClpXP system (**Figure** 1A), consisting of proteins ClpX, ClpP, and SspB in *H. bluephagenesis*, was identified via homologous sequence alignment with corresponding proteins in *E. coli* (Figure 1D). Among these three genes, *clpX* and *clpP* were located in the same operon. Additionally, the *tmRNA* gene was identified, and the SsrA tag sequence was determined to be AANDENYAQGALAA, closely resembling the *E. coli* SsrA sequence (AANDENYALAA).^[^ ^14^ ^]^

To elucidate the function of the ClpXP system, a plasmid harboring *gfp* gene fused with the DNA sequence of wild‐type SsrA at its 3′ end was constructed. A plasmid encoding GFP without the SsrA tag served as the control. Both plasmids were transformed into *H. bluephagenesis* TD01, and the fluorescence intensity (FI) was measured at 12 h intervals over a 48 h culture period in a 60 MM medium. The GFP group exhibited strong and stable fluorescence, whereas the GFP‐SsrA group demonstrated minimal fluorescence, indicating that the identified SsrA tag facilitates rapid degradation of GFP (Figure 1E). The identified wild‐type SsrA tag of *H. bluephagenesis* was named SsrA1.

In *E. coli*, the ClpXP recognition part of SsrA sequence only consists of the last 4 amino acids, which is shorter than SspB recognition part (the first 7 amino acids).^[^ ^14^ ^]^ Therefore, the *sspB* gene was deleted in *H. bluephagenesis* TD01 to eliminate its role in tethering SsrA‐tagged protein to ClpX. This deletion effectively disregards the SspB recognition site in the SsrA tag, shifting the focus to the last four amino acids recognized by ClpXP, thus simplifying the mutation work. To evaluate the role of SspB in cell growth and target protein degradation, the *sspB* gene‐deficient strain *H. bluephagenesis* CYL0119 (TD01Δ*sspB*) was constructed using CRISPR/Cas9. Cell growth and PHB production ability were evaluated through 48 h shake flask experiments. Results showed that cell dry weight (CDW) and PHB content of *H. bluephagenesis* CYL0119 were similar to those of the wild‐type *H. bluephagenesis* TD01 (Figure 1F). These results demonstrated the possibility of reducing the complexity of the mutant library.

### 2.2 Construction of the Mutated SsrA Tags Library

**Figure 2:**
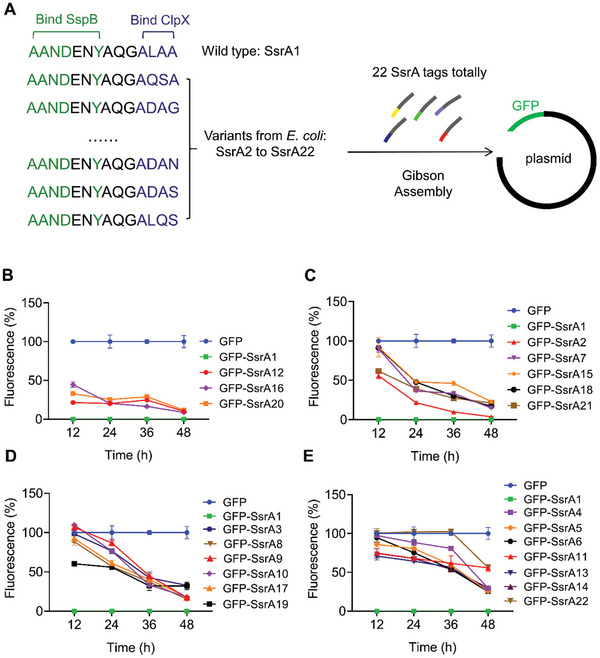
Construction of the mutated SsrA library in *H. bluephagenesis*. A) Construction of mutated SsrA tags based on reported variants in *E. coli*. B) The group of mutated SsrA tags reduces Fluorescence% to lower than 50% before 12 h. C) The group of mutated SsrA tags reduces Fluorescence% to lower than 50% between 12 and 24 h. D) The group of mutated SsrA tags reduces Fluorescence% to lower than 50% between 24 and 36 h. E) Five mutated SsrA tags in the group reduce Fluorescence% to lower than 50% between 36 and 48 h and two mutated SsrA tags in the group maintain Fluorescence% higher than 50%. All data represent the mean of n = 3 biologically independent samples and error bars show s.d.

As mentioned previously, the SsrA tag contains both the SspB recognition sequences and the ClpX recognition sequences (**Figure** **2**A). In an SspB deleted system, ClpX binding sites (ALAA) are the key points to influence protein degradation rates. Following results from *E. coli*, there were 21 mutated SsrA tags chosen for analysis, named SsrA2‐SsrA22 (Figure 2A).^[^ ^13^ ^]^ The amino acids and DNA sequences of all SsrA tags are listed in Tables S3 and S4 (Supporting Information). The DNA sequences of 22 tags were synthesized and respectively inserted into the *gfp* gene before its stop codon “TAA” in plasmid pSEVA321‐GFP, which is able to constitutively express the GFP protein.^[^ ^32^ ^]^

All 22 plasmids were subsequently transformed into *H. bluephagenesis* CYL0119 cultured in 60 MM for 48 h, with a plasmid harboring *gfp* with SsrA1 as the negative control and *gfp* without SsrA as the positive control. Fluorescence intensity (FI) was measured by flow cytometry at 12 h intervals over a 48 h culture period.

The FI of the GFP group and GFP‐SsrA1 group were set to 100% and 0%, respectively. All the FI are shown as Fluorescence% calculated using Equation (1). FI represents the fluorescence intensity of each sample, FI_GFP‐SsrA1_ and FI_GFP_ are the fluorescence intensities of the GFP‐SsrA1 and the GFP groups, respectively.

(1) $$\mathrm{Fluorescence}\%=\left(\mathrm{FI}\;-\;FI_{\mathrm{GFP}-\mathrm{SsrA}1}\right)/\left(FI_{\mathrm{GFP}}-\;FI_{\mathrm{GFP}-\mathrm{SsrA}1}\right)$$

These tags were categorized into four groups based on the half‐life of GFP fused with SsrA tags: 1) very short half‐life (3 tags, whose Fluorescence% is lower than 50% before 12 h); 2) short half‐life (5 tags, whose Fluorescence% is lower than 50% between 12 and 24 h); 3) long half‐life (6 tags, whose Fluorescence% is lower than 50% between 24 and 36 h); and 4) very long half‐life (7 tags, including 5 tags whose Fluorescence% is lower than 50% between 36 and 48 h and 2 tags whose Fluorescence% is still higher than 50% at 48 h) (Figure 2B–E). Thus, via introducing mutations to the ClpX recognition sequence “ALAA”, SsrA tags library with varying degradation rates was successfully constructed.

### 2.3 Morphological Engineering via Fusing Mutated SsrA Tags to MreB

MreB is crucial for maintaining the rod shape of bacteria, and it is essential for bacterial growth.^[^ ^4^, ^5^, ^6^ ^]^ Previous studies showed that deleting *mreB* gene in *E. coli* resulted in spherical cells, but significantly impaired bacterial growth.^[^ ^4^ ^]^ This study aimed to progressively degrade the MreB protein rather than knock out the gene to preserve bacterial growth while achieving the benefits of enlarged spherical cells.

**Figure 3:**
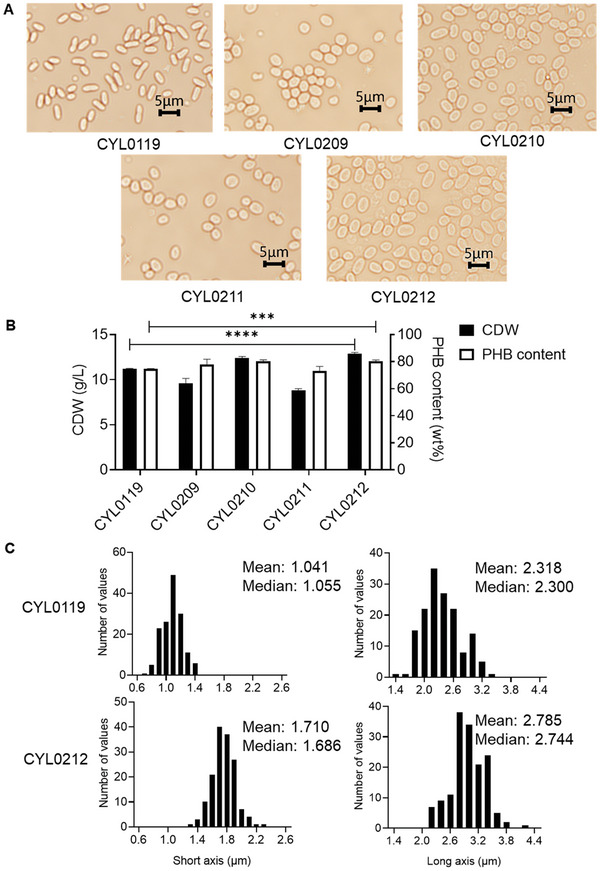
Effects of fusing mutated SsrA tags to MreB. A) Morphology observations of *H. bluephagenesis* CYL0119 (TD01Δ*sspB*) inserted with SsrA5, SsrA16, SsrA17, and SsrA21 before the stop codon of *mreB*, named as *H. bluephagenesis* CYL0209, CYL0210, CYL0211 and CYL0212, respectively. B) Growth (CDW) and PHB content of *H. bluephagenesis* CYL0119, CYL0209, CYL0210, CYL0211, and CYL0212, respectively. C) The frequency distribution histograms of the short axis and the long axis of *H. bluephagenesis* CYL0119 and CYL0212, respectively. All flask data represent the mean of n = 3 biologically independent samples, error bars show s.d. ^***^ indicates *p*<0.001.

Mutated SsrA tags with varying degradation rates were constructed and categorized into four groups (Figure 2B–E). Since the optimal degradation time for MreB was unknown, DNA sequences of SsrA5, SsrA16, SsrA17, and SsrA21 from four groups of different degradation rates, were selected and inserted into the 3′ ends of the *mreB* gene before the stop codon “TGA” in *H. bluephagenesis* CYL0119 using CRISPR/Cas9, resulting in *H. bluephagenesis* CYL0209, CYL0210, CYL0211, and CYL0212, respectively. *H. bluephagenesis* CYL0119 was used as the control. Morphology of all strains was observed under a microscope after culturing in LB60 for 48 h, and results showed that all MreB‐edited strains exhibited a larger shape (**Figure** **3A**). All strains were then cultured in 60 MM for 48 h, with a decreased CDW for all groups except for those inserted with SsrA21 and SsrA16, respectively (Figure 3B). The highest CDW and PHB contents were observed when SsrA21 was fused to MreB in *H. bluephagenesis* CYL0212, resulting in 14.2% and 5% increases in comparison to the control, respectively. The short axis and long axis of *H. bluephagenesis* CYL0119 and CYL0212 were measured based on Figure 3A. Compared with *H. bluephagenesis* CYL0119, the short axis and long axis of *H. bluephagenesis* CYL0212 increased from 1.0 to 1.7 µm and 2.3 to 2.8 µm, respectively (Figure 3C).

### 2.4 Combination of MreB Degradation and MinCD Overexpression

**Figure 4:**
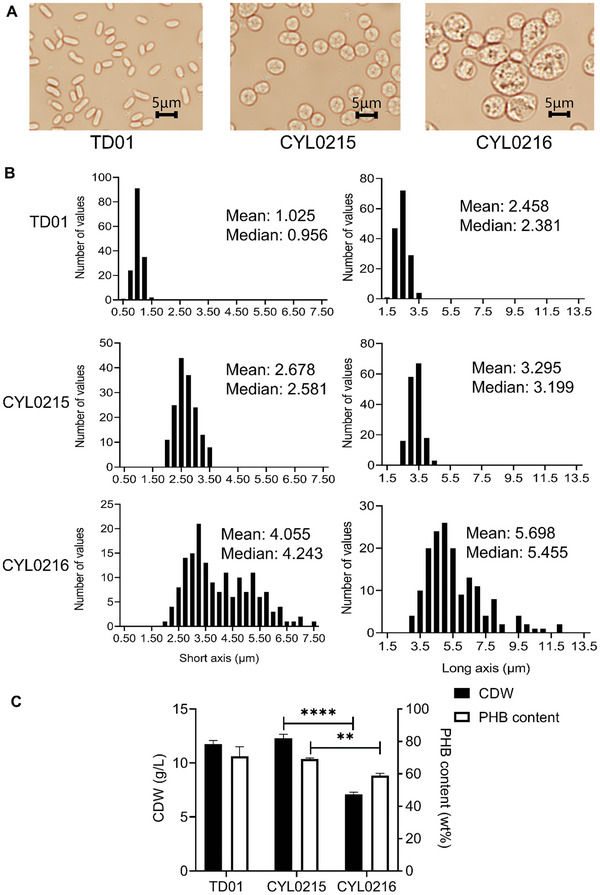
Enhanced sizes of *H. bluephagenesis* CYL0212 (TD01Δ*sspB mreB‐ssrA21*) via over expressing *minCD*. A) Morphology observations of *H. bluephagenesis* TD01, CYL0215 (TD01Δ *sspB*Δenp *mreB‐ssrA21‐*pHbPBC), and CYL0216 (TD01Δ*sspB*Δenp *mreB‐ssrA21* pHbPBC‐P*_phaP1_‐*RBS*_128_‐minCD*), respectively. Δenp indicates that the endogenous plasmid has been deleted in this strain. B) The frequency distribution histograms of the short axis and the long axis of *H. bluephagenesis* TD01, CYL0215, and CYL0216, respectively. C) Growth (CDW) and PHB content of *H. bluephagenesis* TD01, CYL0215, and CYL0216, respectively. All flask data represent the mean of n = 3 biologically independent samples, error bars show s.d. ^**^ indicates *p*<0.01. ^***^ indicates *p*<0.001.

Previous studies showed that controllable overexpression of *minCD* in a modified endogenous plasmid pHbPBC could increase cell length and PHA yield in *H. bluephagenesis*.^[^ ^5^, ^33^ ^]^ Therefore, the endogenous plasmid (enp) in *H. bluephagenesis* CYL0212 was deleted and replaced by the pHbPBC (empty plasmid) and pHbPBC‐P*_phaP1_‐*RBS_128_‐*minCD*, resulting in *H. bluephagenesis* CYL0215 and *H. bluephagenesis* CYL0216. Microscope observation showed that *H. bluephagenesis* CYL0216 exhibited the largest spherical shape (**Figure** **4**A). The short axis and the long axis of *H. bluephagenesis* CYL0216 were 4.0 and 5.7 µm, respectively, which were nearly two times compared with CYL0215 (Figure 4B). However, compared with *H. bluephagenesis* CYL0215, both CDW and PHB content in *H. bluephagenesis* CYL0216 decreased (Figure 4C). Therefore, the strategy of overexpressing *minCD* was not pursued, and there was no need to replace the endogenous plasmid with pHbPBC. Based on these considerations, spherical *H. bluephagenesis* CYL0212, with its large size and normal growth ability, was selected for further studies (Figure 3).

### 2.5 Optimization of PHB Production in *H. bluephagenesis* CYL0212

**Figure 5:**
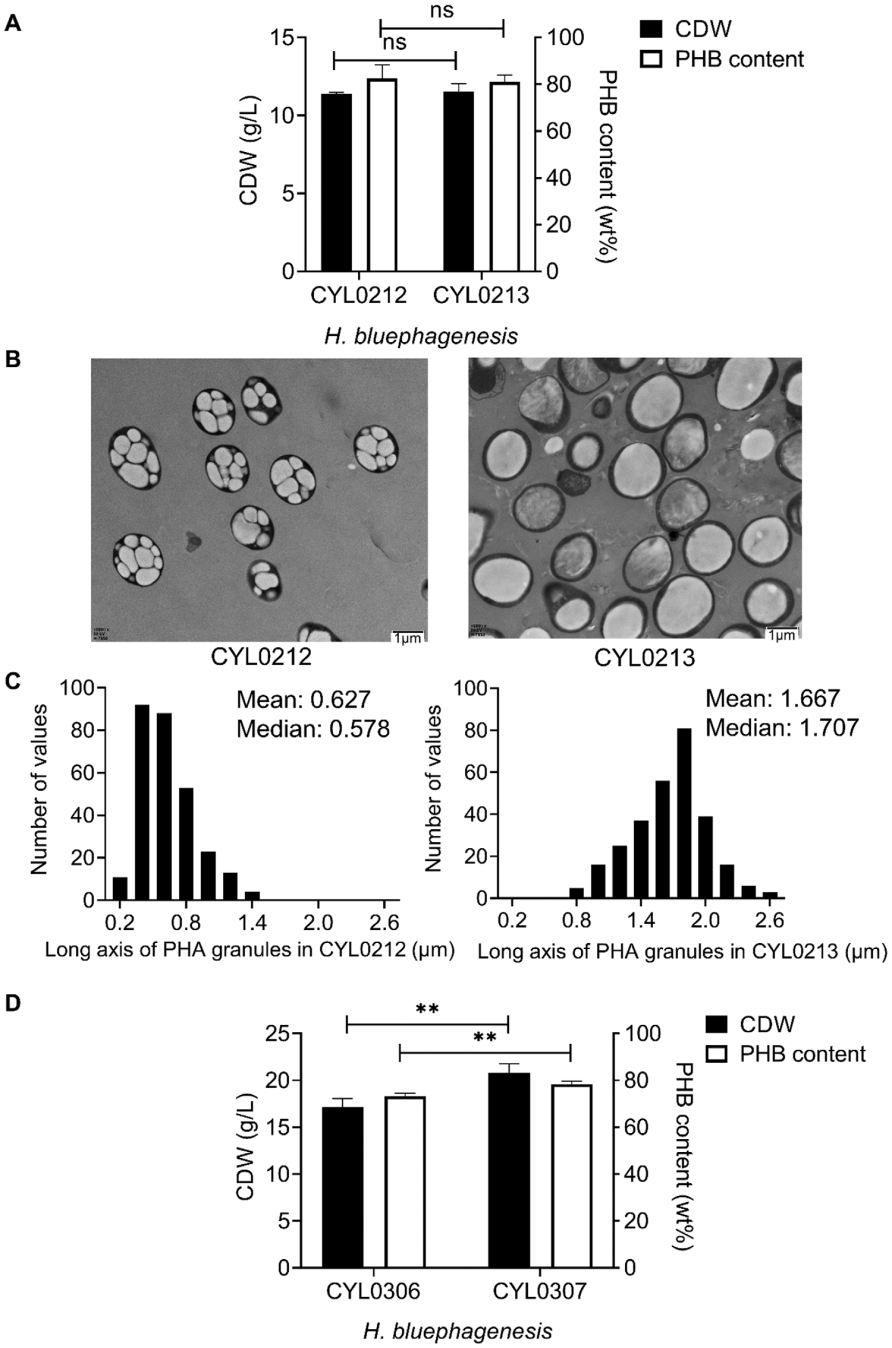
Optimization of PHB production in *H. bluephagenesis* CYL0212 (TD01Δ*sspB mreB‐ssrA21*). A) Growth (CDW) and PHB synthesis by *H. bluephagenesis* CYL0212 (TD01Δ*sspB mreB*‐*ssrA21*) and CYL0213 (TD01Δ*sspB*Δ*phaP1 mreB*‐*ssrA21*). B) TEM studies on *H. bluephagenesis* CYL0212 and CYL0213. C) The frequency distribution histograms of the long axis of PHB granules in *H. bluephagenesis* CYL0212 and CYL0213. D) Growth (CDW) and PHB synthesis under 1 g L^−1^ urea by *H. bluephagenesis* CYL0306 (TD01Δ*sspB*Δ*phaP1*Δenp *mreB*‐*ssrA21* pHbPBC) and *H. bluephagenesis* CYL0307 (TD01Δ*sspB*Δ*phaP1*Δenp *mreB*‐*ssrA21* pHbPBC*‐phaAB_Re_*). All shake flask data represent the mean of n = 3 biologically independent samples and error bars show s.d. ns indicates *p*>0.05. ^**^ indicates *p*<0.01.

PHB granules‐associated proteins phasin (PhaP) cover the surface of PHB granules and prevent small granules from aggregating into large granules.^[^ ^34^, ^35^, ^36^ ^]^ Larger PHB granules formed in *phaP1* knocked out *H. bluephagenesis* benefiting for PHB separation.^[^ ^35^ ^]^ Thus, the *phaP1* gene in *H. bluephagenesis* CYL0212 was deleted, forming *H. bluephagenesis* CYL0213. Both *H. bluephagenesis* CYL0212 and CYL0213 were grown and accumulated PHB similarly (**Figure** **5**A). Compared to multiple small PHB granules in *H. bluephagenesis* CYL0212, a single and much larger PHB granule was observed in *H. bluephagenesis* CYL0213 under transmission electron microscopy (TEM) (Figure 5B). The frequency distribution histograms showed that compared with *H. bluephagenesis* CYL0212, the average long axis of PHB granules in CYL0213 increased from 0.6 to 1.7 µm (Figure 5C).

Since spherical cells have larger volumes for PHB accumulation, the PHB precursor synthesis genes *phaAB_Re_* from *Cuprividus necator* were overexpressed in the modified endogenous plasmid pHbPBC to boost the PHB synthesis flux in *H. bluephagenesis* CYL0213.^[^ ^33^ ^]^ Therefore, the endogenous plasmid (enp) in *H. bluephagenesis* CYL0213 was deleted and replaced by pHbPBC (empty plasmid) and pHbPBC‐*phaAB*_Re_, resulting in *H. bluephagenesis* CYL0306 and CYL0307, respectively.

When cultured in 1 g L^−1^ urea, *H. bluephagenesis* CYL0307 behaved better than it did in 0.5 g L^−1^ urea (Figure S1, Supporting Information), achieving 20.8 g L^−1^ CDW containing 78.4% PHB. In contrast, the control strain CYL0306 reached 17.2 g L^−1^ CDW containing 73.3% PHB (Figure 5D).

### 2.6 Fermenter Scale‐Up Studies in 7, 100, and 5000 L Bioreactors

**Figure 6:**
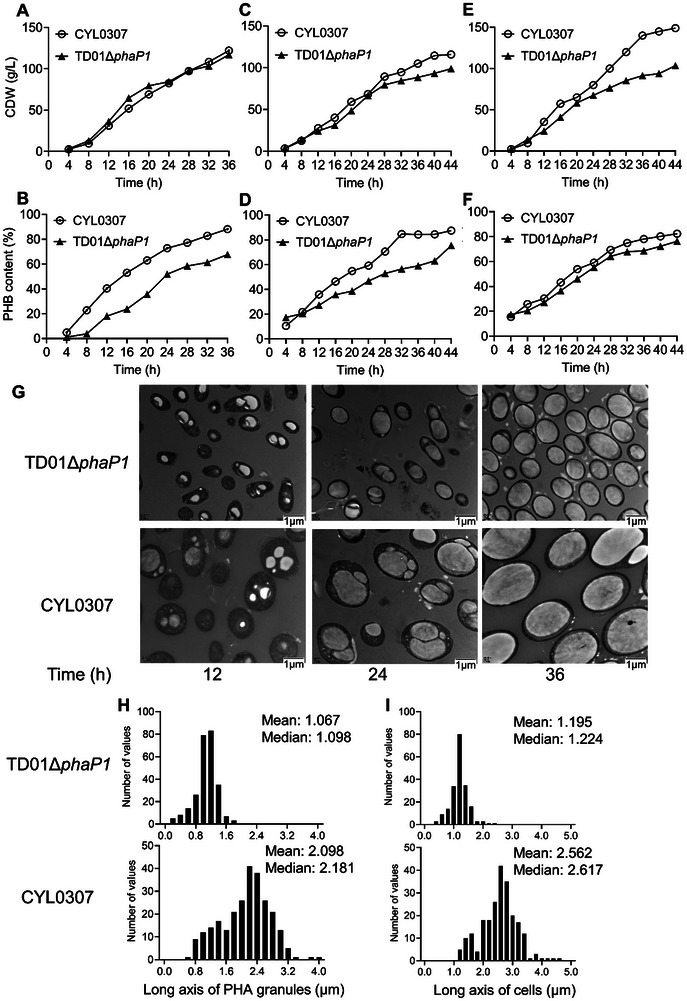
Growth (CDW) and PHB synthesis of *H. bluephagenesis* TD01Δ*phaP1* and CYL0307 in 7, 100, and 5000 L bioreactors, respectively. A,B) Growth (CDW) and PHB synthesis of *H. bluephagenesis* TD01Δ*phaP1* and CYL0307 (TD01Δ*sspB*Δ*phaP1*Δenp *mreB*‐*ssrA21* pHbPBC*‐phaAB_Re_*) in 7 L bioreactors. C,D) Growth (CDW) and PHB synthesis of *H. bluephagenesis* TD01Δ*phaP1* and CYL0307 in 100 L bioreactors. E,F) Growth (CDW) and PHB synthesis of *H. bluephagenesis* TD01Δ*phaP1* and CYL0307 in 5000 L bioreactors. G) TEM observations of *H. bluephagenesis* TD01Δ*phaP1* and CYL0307 grown for 12, 24, and 36 h in 7 L bioreactors, respectively. H) The frequency distribution histograms of the long axis of PHA granules in *H. bluephagenesis* TD01Δ*phaP1* and CYL0307, respectively. I) The frequency distribution histograms of the long axis of cells in *H. bluephagenesis* TD01Δ*phaP1* and CYL0307, respectively.

Based on previous results, *H. bluephagenesis* CYL0307 was selected to be grown in 7, 100, and 5000 L bioreactors to study whether it can be scaled up. All cultivations were conducted under open and unsterile conditions using TD01Δ*phaP1* as the control strain. In 7 L bioreactors with 3 L of fermentation broth, *H. bluephagenesis* CYL0307 achieved 122 g L^−1^ CDW with 88% PHB after 36 h fermentation, while the control strain, *H. bluephagenesis* TD01Δ*phaP1* reached 117 g L^−1^ CDW with only 68% PHB under the same conditions (**Figure** **6**A,B). *H. bluephagenesis* CYL0307 performed better in lab scale‐up.

Subsequently, both *H. bluephagenesis* CYL0307 and TD01Δ*phaP1* were further scaled up for PHB production in 100 and 5000 L bioreactors, respectively. In 100 L bioreactors with 45 L of fermentation broth, *H. bluephagenesis* CYL0307 achieved 116 g L^−1^ CDW containing 87% PHB after 44 h fermentation, while *H. bluephagenesis* TD01Δ*phaP1* reached only 99 g L^−1^ CDW containing 75% PHB under the same conditions (Figure 6C,D). Remarkably, in 5000 L bioreactors with 2500 L of fermentation broth, *H. bluephagenesis* CYL0307 reached 149 g L^−1^ CDW containing 82% PHB with a high glucose‐to‐PHB conversion efficiency of 41% after 44 h fermentation. In contrast, *H. bluephagenesis* TD01Δ*phaP1* reached only 104 g L^−1^ containing 76% PHB under the same conditions (Figure 5E,F).

After 12 h of cultivation, both strains exhibited multiple small PHB granules, as observed under TEM (Figure 6G). After 24 h, small PHB granules grew larger and occupied most of the cellular spaces in both strains. At 36 h, only a single PHB granule was observed in each cell of both strains. Notably, the average long axis of PHB granules in *H. bluephagenesis* CYL0307 reached 2.1 µm and the average long axis of cells was 2.6 µm, both approximately twice that of the control strain *H. bluephagenesis* TD01Δ*phaP1* (Figure 6H,I).

*H. bluephagenesis* CYL0307 has thus proven to be a robust and better strain, capable of scaling up in bioreactors of various sizes compared to its control strain (**Table** **1**).

**Table 1:** Growth (CDW), PHB content, and glucose conversion efficiency by *H. bluephagenesis* TD01Δ*phaP1* and CYL0307 in 7, 100, and 5000 L bioreactors, respectively.

Scale	*H. bluephagenesis*	CDW [g L^−1^]	PHB Content [%]	Glucose Conversion Efficiency [%]
7 L	TD01Δ*phaP1*	117.00	67.60	30.58
	CYL0307	122.05	88.15	35.39
100 L	TD01Δ*phaP1*	98.83	75.38	30.03
	CYL0307	115.85	87.48	32.49
5000 L	TD01Δ*phaP1*	103.50	76.32	32.30
	CYL0307	149.00	82.38	40.51

### 2.7 The Downstream Benefits of *H. bluephagenesis* CYL0307

**Figure 7:**
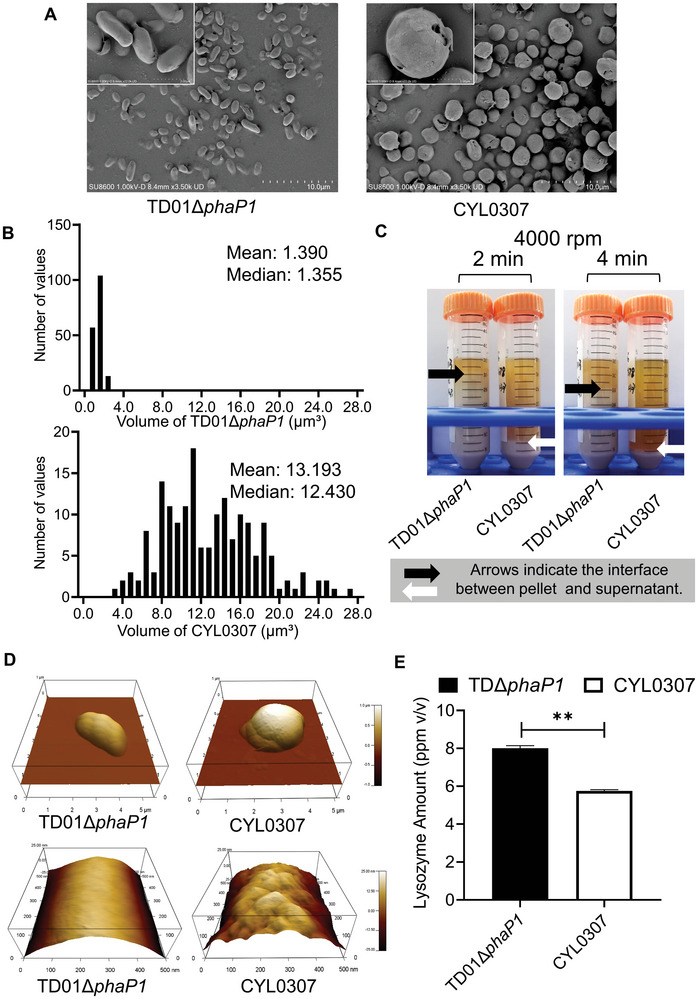
The downstream extraction of *H. bluephagenesis* strains TD01Δ*phaP1* and CYL 0307. A) Morphology observations by SEM of *H. bluephagenesis* TD01Δ*phaP1* and CYL0307 (TD01Δ*sspB*Δ*phaP1*Δenp *mreB*‐*ssrA21* pHbPBC*‐phaAB_Re_*). B) The frequency distribution histograms of cell volumes of TD01Δ*phaP1* and CYL0307, respectively. C) Centrifugation at 4000 rpm of *H. bluephagenesis* TD01ΔphaP1 and CYL 0307 harvested at the end of fermentation in a 7 L bioreactor. The black and white arrows indicate the interface between the pellet and supernatant of *H. bluephagenesis* TD01Δ*phaP1* and CYL0307, respectively. D) Surface roughness observation by AFM of *H. bluephagenesis* TD01Δ*phaP1* and CYL0307, respectively. E) The lysozyme required for cell lysis of *H. bluephagenesis* TD01Δ*phaP1* and CYL0307, respectively. ^**^ indicates *p*<0.01.

Observations under scanning electron microscopy (SEM) revealed that *H. bluephagenesis* CYL0307 formed significantly larger spheres compared to the small rod‐shaped control. (**Figure** **7**A). The transition from rod‐shaped to spherical morphology increased average cell volume from 1.4 to 13.2 µm^3^, which was nine times larger (Figure 7B). Increased cell volume and weight facilitate easier separation by centrifugation. After centrifugation at 4000 rpm for 2 min, it was obvious that *H. bluephagenesis* TDΔ*phaP1* was not completely stratified, while *H. bluephagenesis* CYL0307 is clearly separated into supernatant and pellet. After centrifugation at 4000 rpm for 4 min, the separation was more pronounced (Figure 7C). It is known that for the small rod‐shaped *H. bluephagenesis* TDΔ*phaP1*, centrifugation at 8000 rpm for 10 min is required.^[^ ^34^ ^]^ The reduction in centrifugation speed and centrifugation time decreased equipment requirements and saved energy for the separation process in large‐scale bioprocessing.

Observations under atom force microscopy (AFM) showed that *H. bluephagenesis* CYL0307 exhibited a rougher surface compared to *H. bluephagenesis* TDΔ*phaP1* (Figure 7D). This surface roughness may result from the transition from rod‐shaped to spherical morphology, potentially damaging the outer membrane and facilitating the release of intracellular products. In large‐scale production, the amount of lysozyme used for enzymatic cell lysis is generally adjusted and confirmed based on the final quality of the extracted product. After a comprehensive evaluation, it was found that *H. bluephagenesis* CYL0307 reduced lysozyme usage by 28% (Figure 7E). This reduction in lysozyme use lowers the cost of the extraction process in large‐scale fermentation.

*H. bluephagenesis* CYL0307 has proven to offer benefits in terms of energy and cost savings in downstream extraction.

## 3 Discussion and Conclusion

*H. bluephagenesis* has been recognized as an ideal chassis for the Next‐Generation Industrial Biotechnology conducted under open unsterile conditions.^[^ ^24^ ^]^ However, its small size limited intracellular product accumulation.^[^ ^1^, ^2^, ^3^ ^]^ The cytoskeletal protein MreB plays a key role in maintaining the rod shape of bacteria.^[^ ^7^, ^8^ ^]^ Inhibiting the expression of *mreB* results in the formation of spherical bacteria.^[^ ^2^, ^4^, ^6^ ^]^ Although spherical bacteria provide more cellular space for intracellular accumulation, their growth is severely impaired, leading to reduced production. ^[^ ^2^, ^4^ ^]^

Previous studies indicated that there were three classes of N terminal sequences and two classes of C terminal sequences (degron) recognized by ClpXP involved in many metabolic activities in bacteria, including stalled protein (SsrA), anaerobic reaction (Fnr),^[^ ^37^ ^]^ transposon function (MuA),^[^ ^38^ ^]^ phage infection (λO),^[^ ^39^ ^]^ and stress response (σ^s^).^[^ ^40^ ^]^ These findings highlight the diversity and importance of ClpXP. Unveiling the SsrA tag and ClpXP system in *H. bluephagenesis* helps enhance the understanding of non‐model bacteria.

This study established a novel approach for regulating cell size via controllably degrading cytoskeletal protein MreB. After conducting research on the protein degradation system in non‐model bacteria *H. bluephagenesis*, a mutant library targeting the SsrA tag was constructed for the regulation of essential genes. Specifically, the *sspB* gene was deleted to increase the degradation dependence on the SsrA tag in the engineered ClpXP system (Figure 1B). Then, the SsrA tag sequence was mutated to alter the interaction between SsrA and ClpX so as to adjust the degradation rate of the target protein (Figure 1C). A SsrA tags library was constructed, containing various protein degradation half‐lives in *H. bluephagenesis* to meet different needs (Figure 2). Cytoskeletal protein MreB was chosen to prove the application of this engineered degradation system in morphology engineering. (Figure 3).

When the engineered ClpXP system for progressive degradation of MreB was combined with overexpression of *minCD*, much larger cell sizes were successfully constructed (Figure 4). However, this super‐large cells suffered from reduced growth. To further enhance the PHA production capacity of the enlarged cell volume strain, the MreB degradation strategy was combined with additional modifications, including the knockout of *phaP1* and the overexpression of *phaAB_Re_* (Figures 3 and 5). Notably, the so‐constructed *H. bluephagenesis* CYL0307 demonstrated normal growth with a nine times larger cell volume compared to the starting strain. CYL0307 achieved 116–149 g L^−1^ CDW containing 82–90% PHB in 7, 100, and 5000 L bioreactors, respectively (Figure 6 and Table 1), well proving its capacity for scale‐up with enhanced growth, PHB accumulation and glucose substrate to PHB conversion efficiency.

Large spherical cells of *H. bluephagenesis* CYL0307 not only had more space for intracellular product storage but also exhibited advantages for downstream PHB extraction compared to smaller rod‐shaped cells. Its increased sizes of cells and PHB granules allowed for separation from the cell broth at lower centrifugation speeds with shortened duration (Figure 7). The surface of large *H. bluephagenesis* CYL0307 was weakened, resulting in a 28% reduced lysozyme amount required for cell lysis. The large cells have significantly reduced downstream processing costs, especially for large‐scale industrial production (Figure 7).

Here, a post‐translational control method was established in the non‐model bacterium *H. bluephagenesis* for down‐regulating essential genes. Along with the method, a specific strategy was developed to enlarge cell volume through the controllable degradation of the cytoskeletal protein MreB. Unlike other morphological engineering approaches that negatively impact cell growth or with strong heterogeneity,^[^ ^4^ ^]^ the dynamic degradation of MreB stably enhanced cell volume with normal growth, which is conducive to PHB accumulation and also holds potential for the production of other intracellular products, such as proteins, lipids, starch, polyphosphate, and elemental sulfur.

In summary, this study shows the possibility of engineering enlarged spherical bacterial cells for enhanced accumulation of PHB without reduced cell growth. The so constructed *H. bluephagenesis* CYL0307 has successfully demonstrated its scale‐up feasibility in industries not only for PHB fermentative production but also for advantages in downstream PHB extraction. Moreover, *H. bluephagenesis* CYL0307 as a demonstrated platform that down‐regulates gene expression at post‐translation level has been successfully established. This study provides a new approach to manipulate essential gene expressions without compromising their normal growth at least in their log growth phase. It will help promote the bioproduction of intracellular products.

## 4 Experimental Section

### Strains, Plasmids, and Culture Media

All strains and plasmids used in this study are listed in Tables S1 and S2 (Supporting Information). Plasmids were constructed using either the Gibson Assembly method or the T4 ligation method.^[^ ^41^ ^]^ DNA fragments were amplified by PCR using Q5 DNA polymerase (New England Biolabs Inc.) or Phanta Super‐Fidelity DNA Polymerase (Vazyme Inc.). For cell growth, *E. coli* S17‐1 was cultured in LB10 with 10 g L^−1^ tryptone, 5 g L^−1^ yeast extract and 10 g L^−1^ NaCl, while *H. bluephagenesis* was cultured in LB60 with 10 g L^−1^ tryptone, 5 g L^−1^ yeast extract and 60 g L^−1^ NaCl. For PHB production, *H. bluephagenesis* was cultured in mineral medium 60 (60 MM) with 1 g L^−1^ yeast extract, 60 g L^−1^ NaCl, 0.5 g L^−1^ or 1 g L^−1^urea, 0.2 g L^−1^ MgSO_4_, 10 g L^−1^ Na_2_HPO_4_·12H_2_O, 1.5 g L^−1^ KH_2_PO_4_, 10 g L^−1^ trace element solution I and 1 g L^−1^ trace element solution II. The glucose concentration was adjusted as needed. Antibiotics were added as required: 25 µg L^−1^ chloramphenicol, 50 µg L^−1^ kanamycin, and 100 µg L^−1^ spectinomycin.

### Conjugation

All plasmids were transformed from *E. coli* S17‐1 to the target strain *H. bluephagenesis* via conjugation. Both *E. coli* S17‐1 and *H. bluephagenesis* were cultured for ≈12 h, then transformed to fresh media and grown for an additional 3–4 h to reach an OD_600_ of 0.3–0.5. Both strains were harvested at a 1:1 ratio by centrifugation at a speed of 4000 rpm for 2 min, then washed with fresh LB20 medium, centrifuged again, and suspended together in 50 µL of LB20. The mixture was cultured on LB20 agar plate without antibiotic for 6–8 h at 37 °C. The resulting bacterial lawn was resuspended in 100 µL of LB60 and spread onto LB60 agar plates containing appropriate antibiotics, depending on the plasmid.

### Flow Cytometry Analysis

The samples were prepared by adding 10 µL bacteria culture to 240 µL of PBS. A 20 µL aliquot of each sample was then measured using a flow cytometer (LSRFortessa4, BD, USA). GFP was excited at 488 nm, and signals were detected using the FITC channel (at 420 V), the forward scatter (FSC) channel (at 450 V), and the side scatter (SSC) channel (at 185 V). At least 10 000 cells were analyzed per sample. The mean fluorescence intensity was calculated using FlowJo software (version 10.6.2).

### Shake Flask Studies

Single colonies from the LB60 agar plate were inoculated into 20 mL of LB60 and cultured at 37 °C, 200 rpm for nearly 12 h to produce the first seed culture. Then 200 µL of the first seed culture was transformed to 20 mL of fresh LB60 medium and cultured at 37 °C, 200 rpm for nearly 8 h, reaching an OD_600_ of 3–5 to produce the secondary seed culture. Finally, 2.5 mL of the secondary seed culture was transferred into 50 mL of 60 MM medium in a 500 mL conical flask and incubated at 37 °C, 200 rpm for 48 h. Each experiment was performed in triplicate.

### Analysis of CDW and PHB Contents

A 35 mL sample was collected either after shaking flask cultivation or during fermentation and then harvested by centrifugation at 8000 rpm for 10 min (CR 21GIII, HITACHI, Japan). The pellet was washed once with distilled water, shaken at 2500 rpm for 5 min, and centrifuged again. CDW was calculated by measuring the mass of the sample after lyophilization for 18–24 h. For PHB content, 10–20 mg sample was incubated at 100 °C for 4 h in 2 mL of chloroform and 2 mL of esterification reagent (3% H_2_SO4 and 0.1% benzoic acid in methanol). After cooling to room temperature, 1 mL of distilled water was added for phase separation and extraction. The heavy phase containing PHB was used for gas chromatography analysis (GC‐2014, Shimadzu, Japan). Highly pure PHB from Sigma–Aldrich was used as a standard.

### Observation Under Microscopy

For optical microscopy, 1.5 µL of bacteria cultured in liquid were spread onto a slide and observed. The samples were observed using optical microscopy (IX83, Olympus, Japan).

For atom force microscopy (AFM) studies, mica wave plates were pretreated with 0.01% poly‐L‐lysine overnight. Then 1 mL of cultivated bacteria was harvested by centrifugation at 1500 g for 3 min, washed twice with PBS, and resuspended in 100 µL of PBS. A 10 µL aliquot of the suspension was placed on the pretreated mica plate and incubated at room temperature for 20 min. The plate was then washed twice with deionized water and air‐dried. The samples were examined using AFM (Cypher VRS, Oxford, China).

For scanning electron microscopy (SEM) studies, 1 mL of cultured bacteria was harvested by centrifugation at 4000 g for 5 min, and the supernatant was discarded. The bacterial pellet was then resuspended and fixed in a solution of 2% paraformaldehyde and 2.5% glutaraldehyde at room temperature for 1 h. After fixation, the sample was washed 4 times with 0.1 m phosphate buffer (PB) and subjected to dehydration with ethanol in graded concentrations (50%, 70%, 80%, 90%, 100%, 100%, and 100%) for 15 min each. The sample was then brought to the critical point dryer. Imaging was performed using an SEM SU 8600 (Hitachi, Japan).

For transmission electron microscopy (TEM) studies, 1 mL of bacterial culture was harvested by centrifugation at 4000 g for 5 min, and the supernatant was discarded. The bacterial pellet was resuspended and fixed in 2% paraformaldehyde and 2.5% glutaraldehyde at room temperature for 1 h. Post‐fixation was carried out with 1% osmium tetroxide and 1.5% tetrapotassium hexacyanoferrate trihydrate for 1 h at 22 °C. The sample was dehydrated with ethanol in graded solutions (50%, 70%, 80%, 90%, 100%, 100%, and 100%) for 15 min each, followed by two 15 min treatments with 1,2‐epoxypropane. Gradient infiltration with a mixture of 1,2‐epoxypropane and Epon 812 resin was performed for 8 h (SPI, USA), followed by pure Epon 812 for 2 treatments. Polymerization occurred at 60 °C in an oven. The resin blocks were sectioned using a Leica EM UC7 ultramicrotome (Wetzlar, Germany) into ultra‐thin sections (70 nm), mounted on coated copper grids, and dried. Sections were stained on‐grid with 2% uranyl acetate for 25 min and lead citrate for 5 min. Imaging was carried out under a TEM H‐7650B (Hitachi, Tokyo, Japan) at 80 kV.

The short axis and long axis of PHA granules and cells were measured by ImageJ. For volume calculation, rod‐shaped bacteria were considered as cylinders flanked by two hemispheres, while spherical bacteria were treated as spheres directly.^[^ ^42^ ^]^ All frequency distribution histograms were generated based on the counts derived from a sample size exceeding 150 cells.

### Fermentation Studies in 7, 100, and 5000 L Bioreactor

The preliminary seed culture was prepared similarly to the shake flask method. Then 1 mL of preliminary seed culture was transformed to 100 mL of fresh LB60 in a 500 mL conical flask and cultured at 37 °C, 200 rpm for nearly 10 h until an OD_600_ of 3–5 was reached. The secondary culture, totaling 300 mL, was transferred to a 7 L bioreactor (Bailun, Beijing, China) containing 2.7 L of medium. The final concentrations in the 60 MM medium were adjusted to 5 g L^−1^ urea and 20 g L^−1^ glucose. The pH of the culture medium during fermentation was maintained at ≈8.5. Glucose was supplemented as needed to maintain a concentration of ≈10 g L^−1^. The fed‐batch process was performed under non‐sterile conditions at 37 °C, with 35 mL samples collected every 4 h.

For fermentation in a 100 L bioreactor (Bailun, Beijing, China), the preliminary seed culture was prepared similarly to the shake flask method. Then 10 mL of preliminary seed culture was transformed to 1 L of fresh LB60 in a 5 L conical flask and cultured at 37 °C, 200 rpm for nearly 10 h until an OD600 of 3–5 was reached. The secondary culture, totaling 4 L, was transferred to a 100 L bioreactor containing 41 L of medium. The medium composition and fed‐batch process were the same as those used in the 7 L bioreactors.

For fermentation in the 5000 L bioreactor (PhaBuilder, Beijing, China), the preliminary seed culture and secondary seed culture were prepared in the same manner as for the 100 L fermentation. Then 4 L of secondary seed culture was transformed to 296 L of fresh 60 MM in a 500 L bioreactor for nearly 9 h until an OD600 of 5–6 was reached. Subsequently, the culture (totaling 300 L) was transferred from the 500 L bioreactor into a 5000 L bioreactor containing 2200 L of medium. The medium composition and fed‐batch process were the same as those used in the 7 L bioreactors.

### Cell Lysis and Product Extraction

The fermentation broth was centrifuged to separate bacterial biomass. The biomass was washed with water, re‐centrifuged, and suspended. Subsequently, the cells were heated, lysozyme was added and the mixture was stirred for 1 h. The mixture was centrifuged to obtain the pellet, followed by suspension in water. Protease was added and the mixture was processed with a colloid mill for 1 h. Finally, the mixture was filtered under pressure to form a filter cake, which was dried in the flash‐drying tower. After drying, the product purity, total nitrogen content, and melt mass‐flow rate were measured to assess its quality as described previously.

## Conflict of Interest

The authors declare no conflict of interest.

## Supporting information



Supporting Information

## Data Availability

The data that support the findings of this study are available from the corresponding author upon reasonable request.
