# Supplementary material for: Cell Sizes Matter for Industrial Bioproduction, a Case of Polyhydroxybutyrate
Source: Adv Sci (Weinh). 2025 Feb 18;12(14):2412256. doi: 10.1002/advs.202412256 (PMC11984891; doi:10.1002/advs.202412256)
Supplement: Supplementary file 1 — Supporting Information [file ADVS-12-2412256-s001.docx]

Supporting Information

Cell Sizes Matter for Industrial Bioproduction, A Case of Polyhydroxybutyrate

Yi-Ling Chen, Xu Liu, Li-Zhan Zhang, Ji-Shuai Yang, Wei-Ke Guo, Shuang Zheng, Jia-Le Wang, Fu-Qing Wu, Xu Yan, Qiong Wu, Guo-Qiang Chen*


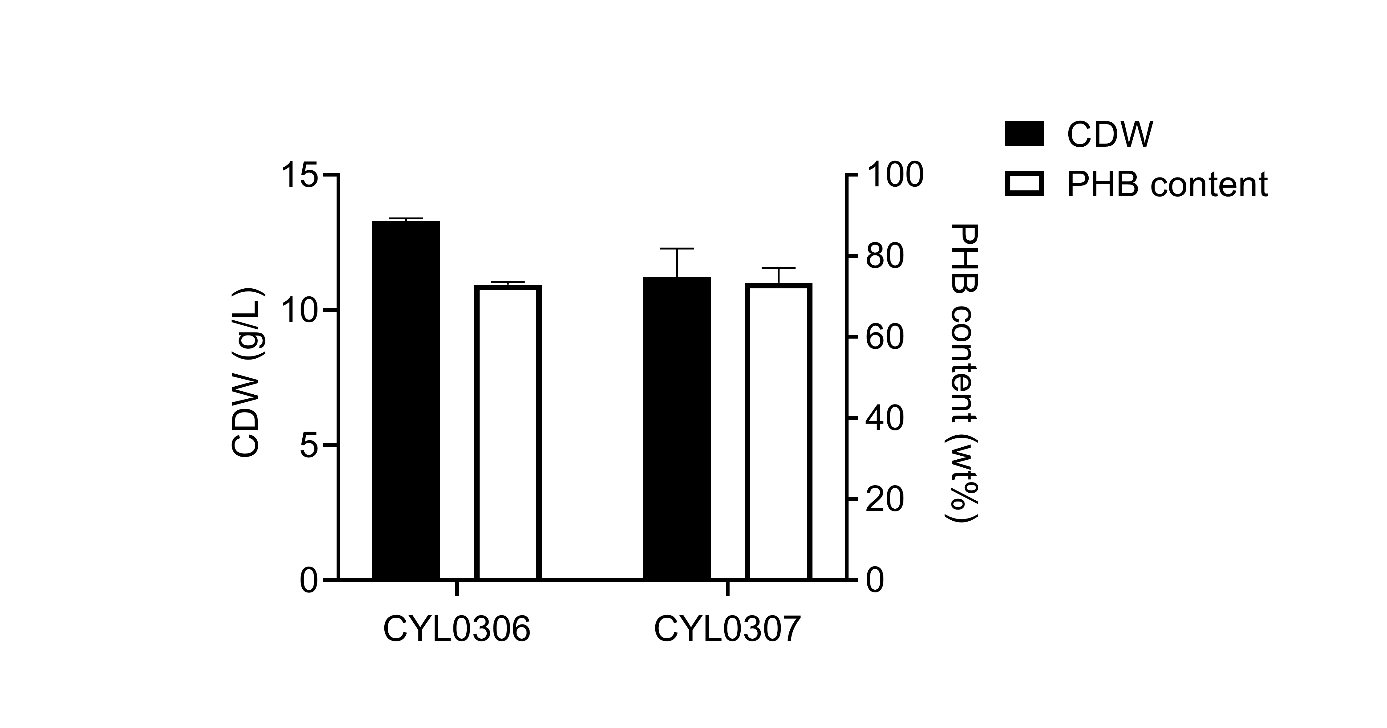


**Figure S1.** Growth (CDW) and PHB synthesis under 0.5 g L^-1^ urea by control strain *H. bluephagenesis* CYL0306 (TD01Δ*sspB*Δ*phaP1*Δenp *mreB*-*ssrA21* pHbPBC) and *H. bluephagenesis* CYL0307 (TD01Δ*sspB*Δ*phaP1*Δenp *mreB*-*ssrA21* pHbPBC*-phaAB_Re_*).

**Table S1.** Microbial strains used in this study.

| Strains | Abbreviation | Description | Source |
| --- | --- | --- | --- |
| *Escherichia coli* S17-1 | S17-1 | *tra* genes of RP4 plasmid integrated on the chromosome; used for conjunction and transform plasmid to *Halomonas bluephagenesis* | [1] |
| *Halomonas bluephagenesis*  TD01 | TD01 | Wild-type strain | [2] |
| TD01Δ*phaP1* | TD01Δ*phaP1* | TD01 deleted *phaP1* gene | [3] |
| TD01Δ*sspB* | CYL0119 | TD01 deleted *sspB* gene | This study |
| TD01Δ*sspB mreB*-*ssrA5* | CYL0209 | CYL0119 with *ssrA5* tag inserted before the stop codon of *mreB* gene | This study |
| TD01Δ*sspB mreB*-*ssrA16* | CYL0210 | CYL0119 with *ssrA16* tag inserted before the stop codon of *mreB* gene | This study |
| TD01Δ*sspB mreB*-*ssrA17* | CYL0211 | CYL0119 with *ssrA17* tag inserted before the stop codon of *mreB* gene | This study |
| TD01Δ*sspB mreB*-*ssrA21* | CYL0212 | CYL0119 with *ssrA21* tag inserted before the stop codon of *mreB* gene | This study |
| TD01Δ*sspB*Δ*phaP1 mreB*-*ssrA21* | CYL0213 | CYL0212 deleted *phaP1* gene | This study |
| TD01Δ*sspB*Δenp *mreB*-*ssrA21* pHbPBC | CYL0215 | CYL0212 replaced endogenous plasmid with empty pHbPBC | This study |
| TD01Δ*sspB*Δenp *mreB*-*ssrA21* pHbPBC*-*P*_phaP1_-*RBS*_128_-minCD* | CYL0216 | CYL0212 replaced endogenous plasmid with pHbPBC*-*P*_phaP1_-*RBS*_128_-minCD* | This study |
| TD01Δ*sspB*Δ*phaP1*Δenp *mreB*-*ssrA21* pHbPBC | CYL0306 | CYL0213 replaced endogenous plasmid with empty pHbPBC | This study |
| TD01Δ*sspB*Δ*phaP1*Δenp *mreB*-*ssrA21* pHbPBC*-phaAB_Re_* | CYL0307 | CYL0213 replaced endogenous plasmid with pHbPBC*-phaAB_Re_* | This study |

**Table S2.** Plasmids used in this study.

| Plasmids | Description | | Source |
| --- | --- | --- | --- |
| pSEVA321 | *trfA* replication origin, *oriT*, Cm^R^ | [4] | |
| pSEVA341 | pRO1600/ColE1 replication origin, *oriT*, Km^R^ and Sp^R^ | [4] | |
| pQ08 | pSEVA321 derivative, *S. pyogenes cas9*, Cm^R^ | [5] | |
| pHbPBC | pHbCP/ColE1 replication origin, *oriT*, *hbpB*/*hbpC*, Km^R^ and Sp^R^ | [6] | |
| pHbPBC*-*P*_phaP1_-*RBS*_128_-minCD* | pHbPBC derivative, P*_phaP1_-*RBS*_128_-minCD*, Km^R^ and Sp^R^ | [7] | |
| pSEVA341-*sspB*-knockout | pSEVA341 derivative, Δ*sspB*, Km^R^ and Sp^R^ | This study | |
| pSEVA321-GFP | pSEVA321 derivative, P*_porin_*_68_-*gfp* | This study | |
| pSEVA321-*gfp-ssrA* tags | pSEVA321 derivative, P*_porin_*_68_-*gfp-ssrA tags* (22 tags totally), Cm^R^ | This study | |
| pSEVA341-*mreB-ssrA* tags-knockin | pSEVA341 derivative, TGA*_mreB_*::*ssrA* tags (4 tags totally), Km^R^ and Sp^R^ | This study | |
| pSEVA341-*phaP1*-knockout | pSEVA341 derivative, Δ*phaP1*, Km^R^ and Sp^R^ | This study | |
| pHbPBC-*phaAB*_Re_ | pHbPBC derivative, *phaAB*_Re_ operon, Km^R^ and Sp^R^ | This study | |

**Table S3.** the amino acid sequences of SsrA tags used in this study.^[8]^

| SsrA Number | Sequence of amino acids |
| --- | --- |
| SsrA1 | AANDENYAQGALAA |
| SsrA2 | AANDENYAQGADAC |
| SsrA3 | AANDENYAQGADAG |
| SsrA4 | AANDENYAQGADAN |
| SsrA5 | AANDENYAQGADAS |
| SsrA6 | AANDENYAQGADAT |
| SsrA7 | AANDENYAQGADAV |
| SsrA8 | AANDENYAQGADCS |
| SsrA9 | AANDENYAQGADKS |
| SsrA10 | AANDENYAQGADSA |
| SsrA11 | AANDENYAQGADSS |
| SsrA12 | AANDENYAQGADTS |
| SsrA13 | AANDENYAQGADVS |
| SsrA14 | AANDENYAQGAHHA |
| SsrA15 | AANDENYAQGAISS |
| SsrA16 | AANDENYAQGAISV |
| SsrA17 | AANDENYAQGALDD |
| SsrA18 | AANDENYAQGALGG |
| SsrA19 | AANDENYAQGALQS |
| SsrA20 | AANDENYAQGALWW |
| SsrA21 | AANDENYAQGAQSA |
| SsrA22 | AANDENYAQGDDAS |

**Table S4.** the DNA sequences of *ssrA* tags used in this study.

| SsrA Number | Sequence of DNA |
| --- | --- |
| SsrA1 | gcggcaaatgacgaaaactacgctcaaggcGCGCTGGCGGCG |
| SsrA2 | gcggcaaatgacgaaaactacgctcaaggcGCGGATGCGTGC |
| SsrA3 | gcggcaaatgacgaaaactacgctcaaggcGCGGATGCGGGC |
| SsrA4 | gcggcaaatgacgaaaactacgctcaaggcGCGGATGCGAAC |
| SsrA5 | gcggcaaatgacgaaaactacgctcaaggcGCGGATGCGAGC |
| SsrA6 | gcggcaaatgacgaaaactacgctcaaggcGCGGATGCGACC |
| SsrA7 | gcggcaaatgacgaaaactacgctcaaggcGCGGATGCGGTG |
| SsrA8 | gcggcaaatgacgaaaactacgctcaaggcGCGGATTGCAGC |
| SsrA9 | gcggcaaatgacgaaaactacgctcaaggcGCGGATAAAAGC |
| SsrA10 | gcggcaaatgacgaaaactacgctcaaggcGCGGATAGCGCG |
| SsrA11 | gcggcaaatgacgaaaactacgctcaaggcGCGGATAGCAGC |
| SsrA12 | gcggcaaatgacgaaaactacgctcaaggcGCGGATACCAGC |
| SsrA13 | gcggcaaatgacgaaaactacgctcaaggcGCGGATGTGAGC |
| SsrA14 | gcggcaaatgacgaaaactacgctcaaggcGCGCACCACGCG |
| SsrA15 | gcggcaaatgacgaaaactacgctcaaggcGCGATCAGCAGC |
| SsrA16 | gcggcaaatgacgaaaactacgctcaaggcGCGATCAGCGTG |
| SsrA17 | gcggcaaatgacgaaaactacgctcaaggcGCGCTGGATGAT |
| SsrA18 | gcggcaaatgacgaaaactacgctcaaggcGCGCTGGGCGGC |
| SsrA19 | gcggcaaatgacgaaaactacgctcaaggcGCGCTGCAGAGC |
| SsrA20 | gcggcaaatgacgaaaactacgctcaaggcGCGCTGTGGTGG |
| SsrA21 | gcggcaaatgacgaaaactacgctcaaggcGCGCAGAGCGCG |
| SsrA22 | gcggcaaatgacgaaaactacgctcaaggcGATGATGCAAGC |

[1] R. Simon, U. Priefer, A. Pühler, *Bio/Technology* **1983**, *1*, 784.

[2] D. Tan, Q. Wu, J. C. Chen, G. Q. Chen, *Metab. Eng.* **2014**, *26*, 34.

[3] R. Shen, Z. Y. Ning, Y. X. Lan, J. C. Chen, G. Q. Chen, *Metab. Eng.* **2019**, *54*, 117.

[4] R. Silva-Rocha, E. Martínez-García, B. Calles, M. Chavarría, A. Arce-Rodríguez, A. de las Heras, A.D. Páez-Espino, G. Durante-Rodríguez, J. Kim, P.I. Nikel, R. Platero, V. de Lorenzo, *Nucleic Acids Res.* **2013**, *41*, D666.

[5] Q. Qin, C. Ling, Y. Zhao, T. Yang, J. Yin, Y. Guo, G.Q. Chen, *Metab. Eng.* **2018**, *47*, 219.

[6] K. Ren, Y. Zhao, G. Q. Chen, X. Ao, Q. Wu, *ACS Synth. Biol.* **2014**, *13*, 61.

[7] S. Zheng, Z. Zhang, P. Jiang, J. Wang, Y. Zheng, K. Ren, L. Zhang, X. Yan, X. Liu, G. Q. Chen, *Chem. Eng. J.* **2024**, *489*, 151413.

[8] K.L. Griffith, A.D. Grossman, *Mol. Microbiol.* **2008**, *70*, 1012.
